# GPT-agents based on medical guidelines can improve the responsiveness and explainability of outcomes for traumatic brain injury rehabilitation

**DOI:** 10.1038/s41598-024-58514-9

**Published:** 2024-04-01

**Authors:** Li Zhenzhu, Zhang Jingfeng, Zhou Wei, Zheng Jianjun, Xia Yinshui

**Affiliations:** 1 Radiology Department, Ningbo NO.2 Hospital, Ningbo, 315211 China; 2 Department of Neurosurgery, Ningbo NO.2 Hospital, Ningbo, 315211 China; 3 Faculty of Electrical Engineering and Computer Science, Ningbo University, Ningbo, 315211 China

**Keywords:** Large language model, Generative pre-trained transformer, Medical guidelines, Traumatic brain injury, Rehabilitation, Health care, Medical research, Neurology, Mathematics and computing

## Abstract

This study explored the application of generative pre-trained transformer (GPT) agents based on medical guidelines using large language model (LLM) technology for traumatic brain injury (TBI) rehabilitation-related questions. To assess the effectiveness of multiple agents (GPT-agents) created using GPT-4, a comparison was conducted using direct GPT-4 as the control group (GPT-4). The GPT-agents comprised multiple agents with distinct functions, including “Medical Guideline Classification”, “Question Retrieval”, “Matching Evaluation”, “Intelligent Question Answering (QA)”, and “Results Evaluation and Source Citation”. Brain rehabilitation questions were selected from the doctor-patient Q&A database for assessment. The primary endpoint was a better answer. The secondary endpoints were accuracy, completeness, explainability, and empathy. Thirty questions were answered; overall GPT-agents took substantially longer and more words to respond than GPT-4 (time: 54.05 vs. 9.66 s, words: 371 vs. 57). However, GPT-agents provided superior answers in more cases compared to GPT-4 (66.7 vs. 33.3%). GPT-Agents surpassed GPT-4 in accuracy evaluation (3.8 ± 1.02 vs. 3.2 ± 0.96, *p* = 0.0234). No difference in incomplete answers was found (2 ± 0.87 vs. 1.7 ± 0.79, *p* = 0.213). However, in terms of explainability (2.79 ± 0.45 vs. 07 ± 0.52, *p* < 0.001) and empathy (2.63 ± 0.57 vs. 1.08 ± 0.51, *p* < 0.001) evaluation, the GPT-agents performed notably better. Based on medical guidelines, GPT-agents enhanced the accuracy and empathy of responses to TBI rehabilitation questions. This study provides guideline references and demonstrates improved clinical explainability. However, further validation through multicenter trials in a clinical setting is necessary. This study offers practical insights and establishes groundwork for the potential theoretical integration of LLM-agents medicine.

## Introduction

Based on date provided by the World Health Organization, traumatic brain injury (TBI) is the third leading cause of death globally^1^, accounting for nearly half of all injury-related deaths worldwide^1,2^. Moreover, TBI is a major cause of acquired disability worldwide; however, effective treatment methods are scarce^3^. Brain trauma can lead to head injuries, skull fractures, brain tissue damage, and, in severe cases, coma, memory loss, and cognitive impairment. Owing to the limited regenerative capacity of the nervous system, the rehabilitation of patients with brain trauma is a lengthy process^3^.

Recently the use of artificial intelligence (AI) to provide personalized medical services for clinical brain rehabilitation has gained significant attention^4^. AI offers the advantage of providing prompt diagnostic and therapeutic recommendations for brain rehabilitation. An emerging area of research is the use of Large Language Models (LLM) as a tool for rehabilitation support, which has gained traction in a variety of fields, including chronic pulmonary disease^5^, rehabilitation education^6^, and physical and skeletal rehabilitation^7,8^. Despite advancements in LLM, this technology has limitations, including issues with accuracy and comprehensiveness^9,10^. LLM may also generate “Hallucinations” ^11,12^, making them unsuitable for providing professional medical advice. Moreover, the lack of explainability^13,14^ of the output results makes it difficult for doctors and patients to establish trust when interacting with a “robotic system”.

In the field of GPT technology, the use of agents is considered the latest approach for tackling complex problems^15,16^. This approach has demonstrated exceptional performance in fields such as programming^17^, gaming^18^, and even complex computer tasks^19^. However, the application of this agent in the medical field remains in the nascent stage. This study therefore aimed to explore the use of an agent technology based on medical guidelines that can provide responses to user inputs. Simultaneously, relevant content from medical guidelines were output within the responses to enhance the explainability of the results.

This study comprised a comparative analysis of the responses between direct GPT-4 and GPT-agents (constructed based on guidelines). A set of brain rehabilitation questions was selected from the doctor-patient Q&A database for assessments. The primary endpoint was a better answer, whereas the secondary endpoints included accuracy, completeness, explainability, and empathy.

## Results

### ChatGPT-agents question-answering system

**Figure 1:**
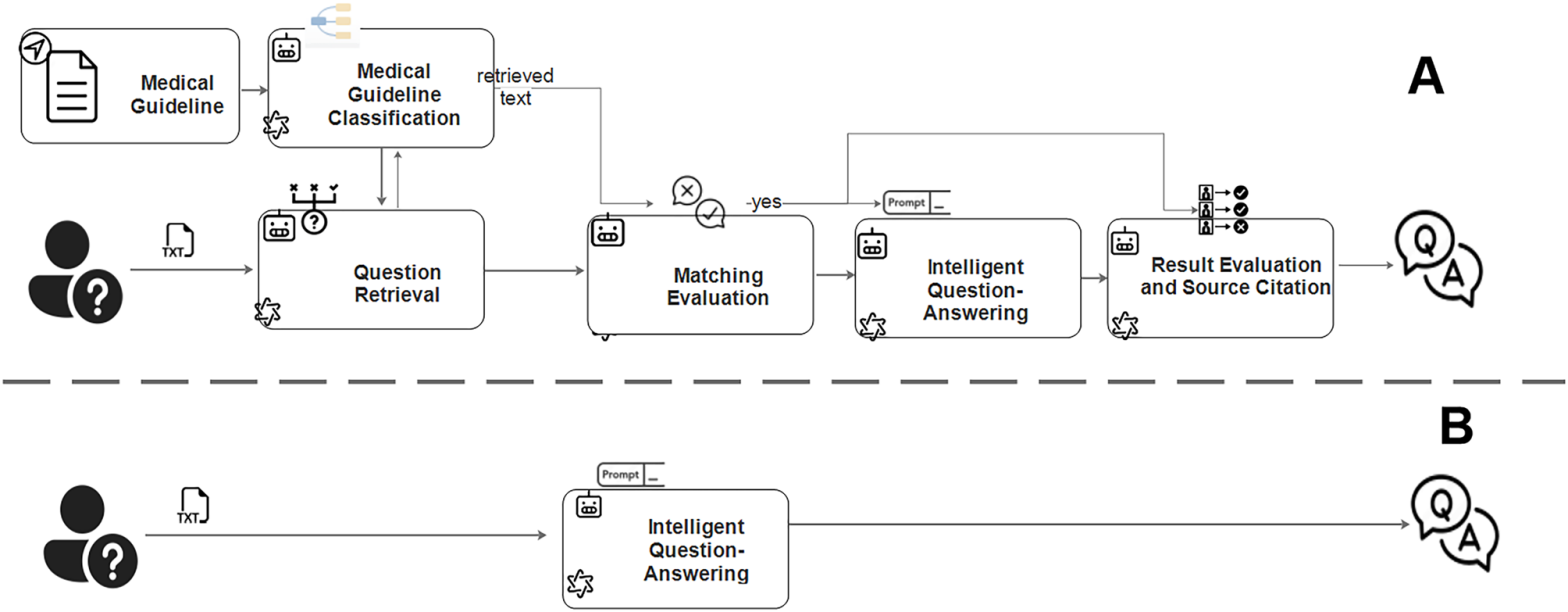
The flowchart illustrates two processesThe flowchart of the GPT process. (**A**) Represents the GPT-Agents based on medical guidelines(group GPT-Agents); (**B**) the direct use of GPT-4(group GPT-4).

**Figure 2:**
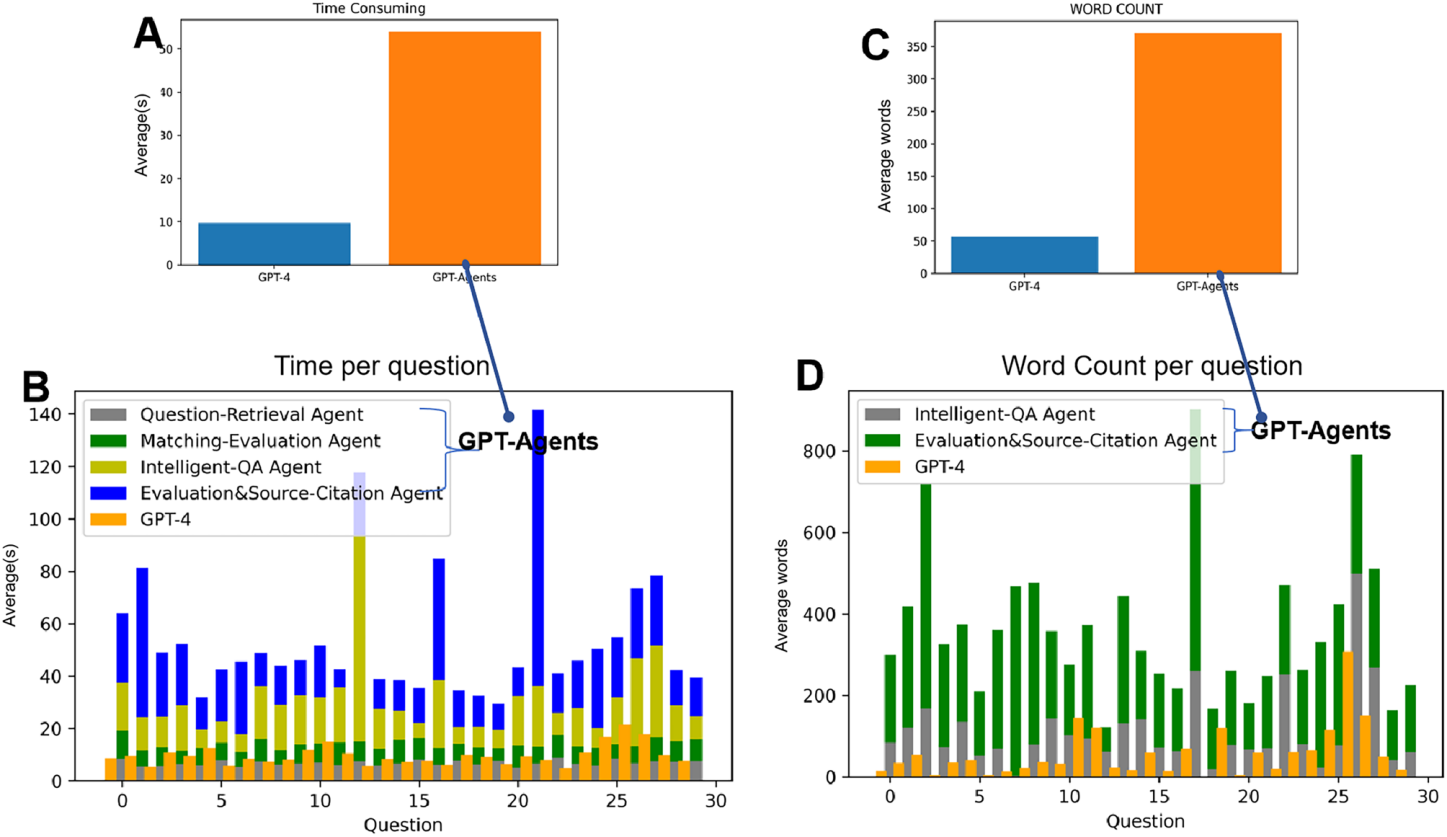
The Time Consumption and Word Count. (**A**, **B**)Time Consumption: the GPT-Agents required more time to answer questions, with an average response time of 54.05 s, whereas GPT-4(direct) takes 9.66 s. (**C**, **D**) The Word Count: GPT-Agents generate more words, with an average word count of 371, whereas GPT-4 produces fewer words with an average count of 57. The "Results Evaluation and Source Citation" was the most time-consuming and word count.

Thirty random questions (Supplementary Table 2) were answered, and it was observed that GPT-agents took significantly longer to respond than GPT-4 (54.05 vs. 9.66 s per question). The “Results Evaluation and Source Citation” agent had the longest response time (Table 1, Fig. 1). Regarding word count, GPT-4 answered in an average of 57 words, which was significantly fewer than the average of 371 words for GPT-agents (Fig. 2).

**Table 1:** The Agents And Function.

index	Agent	Function	Execution State and Input Object
1	Medical Guideline Classification	Conduct clustering analysis of the guidelines, extract the main topics and subtopics of each section, record the textual content under each subtopic, and then save all this information in the form of lists and dictionaries for future retrieval	Run Only Once When Extracting Guidelines
2	Question Retrieval	Search the input question within the subtopics, providing the question and the relevant textual content from the medical guideline as output by the primary agent	Participate in loops, process question, and categorize guidelines
3	Matching Evaluation	Verify the relevance between the question and the text content; if there is a match, output the content; otherwise, output "No," indicating that no relevant content was found in the guideline	Participate in loops, process question and guideline text content
4	Intelligent Question-Answering(QA)	Synchronously input the user's question and the corresponding topic-related text content into the GPT-4 model to generate the answer to the question	Participate in loops, process question and guideline text content
5	Results Evaluation and Source Citation	Firstly, it evaluates the accuracy of the generated answer by comparing it with the contents of the guidelines. Ultimately, it should produce the final response along with the relevant guideline content that corresponds to this response	Participate in loops, process answer and guideline text content

## Evaluation results

**Figure 3:**
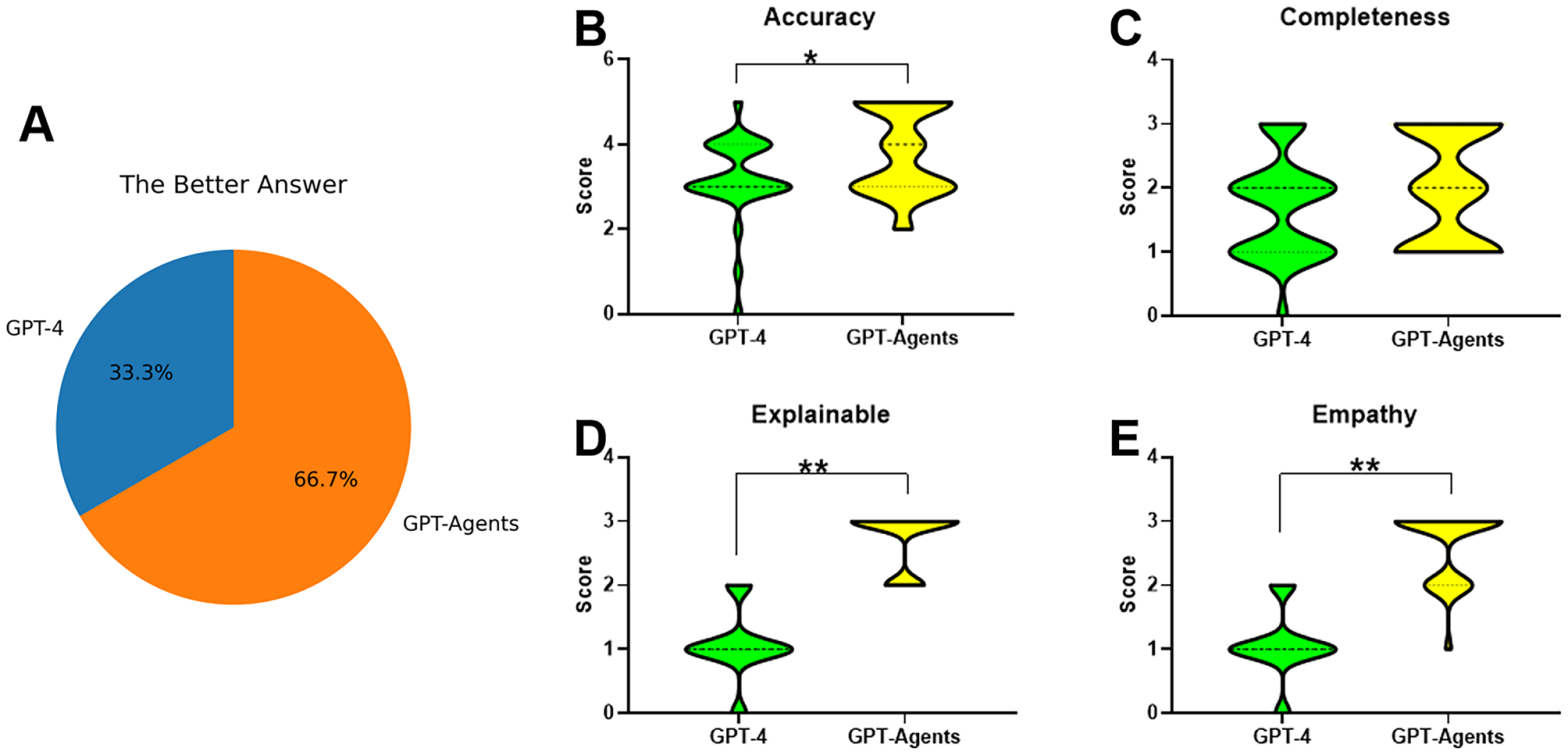
The Evaluation of answer. (**A**) The GPT-Agents was the better one for 66.7% of the answers. (**B**) Accuracy Evaluation: GPT-Agents demonstrated higher accuracy compared to direct GPT-4. (**C**) Completeness Evaluation: Both models showed incompleteness. (**D**) Explainability Evaluation: GPT-Agents exhibited significantly better results than GPT-4. (**E**) Empathy Evaluation: GPT-Agents showed higher empathy compared to GPT-4.

Three evaluators assessed the responses to 30 random questions (Supplementary Table 2). Based on the evaluation results, GPT-agents was found to have provided superior answers in most cases (*n = *20, 66.7%) compared to GPT-4 (*n = *10, 33.3%). Chi-square analysis revealed that GPT-agents significantly outperformed the GPT-4 group (χ^2^ = 6.667,* p* = 0.0098). Further analysis of accuracy evaluation, revealed that the guideline-based GPT-agents (3.8 ± 1.02) outperformed GPT-4 (3.2 ± 0.96, *p* = 0.0234). However, completeness evaluation showed that both models showed incomplete answers, with no significant difference (2 ± 0.87 vs. 1.7 ± 0.79, *p* = 0.213). However, in terms of explainability and empathy evaluation, the GPT-agents performed significantly better than GPT-4 (Table 2, Fig. 3).

**Table 2:** The evaluation results.

	GPT-Agents	GPT-4	*p*
Better Answer (*n = *30)	20	10	0.0098
Accuracy	3.80 ± 1.02	3.20 ± 0.96	0.023
Completeness	2.00 ± 0.87	1.70 ± 0.79	0.213
Explainability	2.79 ± 0.45	1.07 ± 0.52	< 0.001
Empathy	2.63 ± 0.57	1.08 ± 0.51	< 0.001

In the response analysis, when faced with information not covered in the guidelines, GPT-agents explicitly indicated “unclear”, instead of fabricating conflicting content with the guidelines (Supplementary Table 2). In the evaluation section of the results, the GPT-agents explicitly indicated whether the answers were correct, and the specific content from the guidelines (Table 3).

**Table 3:** Few questions and answers.

Index	Question	GPT-4	GPT-Agents	
			Answer	Evalue and Source Citation
1	When is it appropriate to intervene in a brain trauma patient with hyperbaric oxygen therapy?	Within 24–48 h after brain injury	For patients with severe traumatic brain injury with stable vital signs, no active intracranial hemorrhage, no untreated brain hernia, no external ventricular drainage, no severe lung injury, and no cerebrospinal fluid leakage, hyperbaric oxygen therapy should be performed early. The earlier the start of HBO, the better the effect	1. Evaluation of whether it is correct and appropriate: Yes, the answer is correct and appropriate 2. The original text of the relevant knowledge in the guidelines corresponding to the solution to the problem: {For patients with severe traumatic brain injury with stable vital signs, no active intracranial hemorrhage, untreated brain herniation, external ventricular drainage, severe lung injury and cerebrospinal fluid leakage, hyperbaric oxygen therapy should be performed early. The results showed that HBO can significantly increase brain tissue oxygen tension, improve brain tissue metabolism and reduce intracranial pressure. At the same time, HBO can significantly reduce mortality and improve functional prognosis at 6 months. No lung and brain tissue oxygen poisoning was found, and the earlier the HBO started, the better the effect.}
2	What if the patient can't read?	Recommend evaluation and treatment by a speech-language pathologist, which may include speech therapy and use of assistive reading devices. Cognitive training and physical rehabilitation may also be considered	In cases where the patient is unable to read, the following methods can be tried: 1. Use pictures and symbols to communicate 2. Use non-verbal communication methods, such as body language or facial expressions 3. Use electronic voice synthesis devices to help the patient express themselves 4. Try music therapy, especially melodic intonation therapy 5. If possible, try physical therapies such as transcranial direct current stimulation (tDCS) 6. Drug therapy and acupuncture may also be helpful. Please proceed under the guidance of a doctor	1. Evaluation of whether it is correct and appropriate: Yes, the answer is correct and appropriate 2. The original text of the relevant knowledge in the guidelines corresponding to the solution to the problem: - "The basic treatment methods for aphasia can be divided into two categories according to the treatment goals: one category aims to improve language function, including: Schuell stimulation method, blocking removal method, melody therapy; the other category aims to improve daily communication skills, including communication promotion method, compensation method training." - "The training methods for aphasia treatment include: individual training, self-training, group training and family training." - "Music therapy: There are many music therapies that can be used to treat aphasia. The most prominent is the melody intonation therapy of Massachusetts Institute of Technology. This therapy includes two main parts of music therapy: melody intonation and rhythmic typing, and finally to phrase repetition." - "Forced induction therapy for aphasia: Forced induction for the treatment of aphasia is based on 3 principles: ① Short-term use of intensive exercise is more effective than long-term, lower-frequency exercise. ② Patients can be forced to use intensive exercises that they usually avoid. ③ Treatment focuses on relevant behaviors in daily life. Many studies have shown that forced induction therapy is effective, and further studies are needed to expand the sample size." "Transcranial direct current stimulation (tDCS): A systematic analysis showed that although the mechanism by which tDCS can enhance aphasia training is still unclear, studies have shown that tDCS is effective in treating aphasia patients. Another meta-analysis showed that speech rehabilitation training combined with tDCS can improve the naming ability of aphasia patients. In addition, aphasia can also be treated with medication and acupuncture."
3	Can patients with deep vein thrombosis in the lower extremities still undergo rehabilitation training?	Yes	I am sorry, I do not have any relevant reference content. I suggest you consult a doctor or a professional rehabilitation therapist for the most accurate information. Please note that any medical advice should be provided by a suitably qualified healthcare professional	1. Evaluation of correctness and appropriateness: The response content is appropriate. Since no relevant reference content was found, it is correct to suggest consulting a doctor or a professional rehabilitation therapist. In addition, it is also very important to emphasize that any medical advice should be provided by a qualified medical professional 2. The original text of the relevant knowledge in the guidelines corresponding to the solution of the problem: I'm sorry, I couldn't find any relevant reference content. I suggest you consult a doctor or a professional rehabilitation therapist to get the most accurate information. Please note that any medical advice should be provided by a qualified medical professional. This is all the relevant information I could find. If you need more information, I suggest you contact a healthcare professional directly. They can provide more specific and detailed information

## Discussion

In this study, medical guidelines^20^ and agents based on GPT-4 were used to answer questions related to TBI rehabilitation. This system automatically evaluates the correctness of the answers, simultaneously providing relevant content from the medical guidelines to enhance explainability. The evaluation revealed that the responses generated by the guideline-based GPT-agents performed better in terms of accuracy, explainability, and empathy than those obtained by directly querying GPT-4.

Brain rehabilitation is a comprehensive and lengthy treatment process involving a variety of aspects including physical therapy, speech therapy, cognitive training, and psychological support^21,22^. The LLM acquires knowledge from various professional disciplines during training, making it highly suitable for assisting with brain rehabilitation.

Currently, the LLM has demonstrated potential in the medical field^23,24^, owing to its powerful natural language processing and generation capabilities^25,26^. However, the direct use of LMM is still limited by certain challenges, such as inaccurate responses or the generation of hallucinations. Agents based on LLM for complex task processing have shown significant advantages. For example, humans typically perform autonomous programming or automation of certain real-world tasks using computers or smartphones. Agents can also be employed in medical tasks such as for dermatological patient-doctor conversations and assessments^27^. The GPT-agents constructed in this study involved multiple API calls, which results in the generation of lengthier answers, but can increase the response time. Overall, it was found that the GPT-agents had an extended response time compared to GPT-4, but could still provide answers within an average range of 1–2 min, generating an output with a word count between 300 and 700 words (in Chinese). This speed is acceptable for clinical counseling, as it is much shorter than the real-world waiting time in hospitals for treatment.

Traditional direct question-answering systems such as ChatGPT have been found to be limited potential issues related to accuracy^28,29^ and the generation of hallucinatory responses for medical queries^30,31^. Medical guidelines and expert consensus thus serve as the cornerstone of clinical practice. GPT-4 has powerful summarization capabilities^29^, making it a potential tool for guideline classification. In the present study, we observed that after inputting guideline information into the GPT-4, its medical role was significantly activated, leading to improved response accuracy. We further found that the inclusion of guidelines did not directly restrict the agents’ responses. Overall, our GPT-agents could provide suggestions during result evaluation, which offers an alternative when there is no answer available based on the guidelines.

Several studies have previously attempted to improve the accuracy and completeness of LLM by including prompt engineering, fine-tuning, and retraining^29,32^. Considering the high cost of fine-tuning and retraining, this study focused instead on prompt engineering techniques. By utilizing guideline-based agents to process the guidelines and input them as prompts to the GPT, the accuracy of the agents’ responses improved significantly. This improvement could be attributed to the prompt use of medical guidelines, which better set the context and cultural positioning of GPT. Guidelines are commonly modified to suit the specific healthcare environments in a particular region. Thus, different healthcare environments and conditions may implement slightly different approaches for the same medical issue. For example, Traditional Chinese Medicine is often incorporated into medical guidelines and consensus in China^20^. This study followed a logical chain of thinking, incorporating knowledge from medical guidelines, and employed multiple evaluative agents to assess the questions and answers. We believe that providing professional medical guidelines and utilizing evaluative agents are superior strategies for enhancing response quality.

Completeness is defined as the accumulation of experience in long-term clinical work involving insights and reflections on multiple dimensions of illness. In the present study, we found that both GPT-agents and GPT-4 were lacking in terms of completeness, indicating that their ability to answer medical questions is still in the early stages of development. Further research should explore whether combining fine-tuned teleology can improve completeness.

Explainability is an important criterion when evaluating the current use of AI in medicine^14,33^. Because of their large number of parameters, LLMs are inherently difficult to explain. In the present study, the explainability of the results was assessed by referencing the original text of the guidelines. After the answer was evaluated as “correct” or “incorrect”, the related original text of the referenced guideline content was output by the final agent. This significantly increases the explanatory power of the results.

Patients with brain injury often require a lengthy recovery period, and rely on their families for reintegration into society. Empathy can help family members to understand and motivate patients, thus boosting their confidence in treatment. The GPT-4 itself seems to have an advantage over clinical doctors in terms of empathy^34,35^. In the present study, we found that GPT-agents had significantly enhanced empathy compared to the base GPT-4. This may be attributed to the inclusion of more medical information, which provided the GPT with more precise positioning and allowed it to generate words associated with empathy.

Although this study found that GPT-agents based on medical guidelines could significantly improve medical responses, there are still some limitations which should be considered. First, the use of GPT-agents results in an increase in the cost time. Overall, we found an average increase of 1 min in response time for GPT-agents in our study. However, this may be affected by different areas and Internet environments. Secondly, there is the issue of incomplete answers. Clinical practice is complex and involves multiple disciplines. However, no single guideline can adequately address these complex clinical issues. Guidelines are constantly evolving, and may not always align with the most advanced treatment approaches. As such, these guidelines must be critically evaluated. Incorporating a wide and non-duplicate summary guideline can help to overcome this problem. Third, this study did not employ random double-blinding owing to the inclusion of guideline references in the GPT-agents’ responses, making it impossible to implement blinding on assessors, which could have led to subjectivity in the results. Finally, the actual medical environments in hospitals are complex and variable, involving individual patient situations, medical histories, and symptoms. Additionally, ethical and medical regulations differ across regions. ChatGPT may not have fully considered these factors when answering questions, thus limiting the applicability of its responses. As such, when using the GPT, healthcare professionals and clinical teams must maintain professional judgment, integrate GPT responses with specific patient contexts, and develop the best diagnosis and treatment plans accordingly.

In future research, optimization could be continued through several approaches. First, it will be necessary to further refine the foundational large models, particularly by upgrading them to multimodal models. This is crucial, as many patients with clinical brain injury may not be able to complete typing or speaking tasks. Utilizing various input modes (such as voice and images) can help to broaden accessibility. Second, further studies should explore whether agents based on medical guidelines exhibit common patterns in other conditions, such as rare diseases or critical illnesses. It is essential to determine whether employing guideline-based agents can enhance the responses of LLMs. Finally, as various diseases and medical guidelines intersect, research on recommendation algorithms will be necessary. This algorithm should accurately assess and rank diverse search contents, discerning patients' true intentions, as different diseases involve varying guidelines, and a single condition may have multiple treatment guidelines.

Despite these limitations, our research showed that GPT-agents that rely on medical guidelines hold significant promise for various medical applications. By integrating evidence-based guidelines, these agents can utilize the wealth of knowledge and expertise accumulated through extensive clinical practice and research. This integration not only improves the reliability of the generated responses, but also ensures their alignment with established medical standards and best practices.

Overall, the results of this study showed that GPT-agents have enhanced the accuracy and empathy of responses to TBI rehabilitation questions. This study provides guideline references and demonstrates improved clinical explainability. Compared to the direct use of GPT-4, GPT-agents based on medical guidelines showed improved performance, despite the slight increase in response time. With advances in technology, this delay is expected to be minimized. However, further validation through multicenter trials in a clinical setting is necessary. Overall, this study offers practical insights and establishes the groundwork for the potential theoretical integration of LLM-agents in the field of medicine.

## Methods

This study employed a cross-sectional, non-human subject research design. A flowchart of the study design is shown in Fig. 1. As this study did not involve human or animal participants, and ChatGPT/OpenAI could freely access Kaggle.com via the API, Ethical Committee Approval was not required.

Several LLM are currently available; online models include Google's Bard, Microsoft's Bing, Baidu's Wenxin Yiyan, IFLYTEK's Spark, and OpenAI's GPT-series, among others. Offline deployable options include lama and chatglm. Given the popularity of GPT-4 among our research team, GPT-4 was chosen as the foundational model.

In the present study, Multiple agents were constructed using GPT-4, including "Medical Guideline Classification”, “Question Retrieval”, “Matching Evaluation”, “Intelligent Question-Answering”, and “Results Evaluation and Source Citation” (Fig. 1) . The knowledge for the agents was derived from expert consensus or guidelines on brain injury rehabilitation from China.

### Design of guideline-based ChatGPT-agents (GPT-agents)

Guideline-based GPT-agents were designed based on GPT-4. The primary objective of an intelligent agent is to retrieve and provide word suggestions as answers. An evaluation was introduced for each of the steps mentioned above, resulting in five intelligent agents (Table 1). The first agent was responsible for the clustering analysis of the guidelines, extracting the topics and subtopics of each section, and then saving all of these extracted topics for later reference and retrieval. The second agent searched the inputted question within the subtopics, and the output was the question + the related content of medical guideline from the first agent. The third agents performed a “Matching Evaluation,” to check whether the question and the content were relevant. The fourth agent was question-answering agent which synchronously input the user's question and corresponding topic-related content into the GPT-4 model to generate the answer to the question. Finally, the fifth agent performed two functions: firstly, it evaluated the accuracy of the generated answer by comparing it with the contents of the guidelines, and secondly it produced the final response along with the relevant guideline content that corresponding to this response (Fig. 1A).

The program was deployed on the Kaggle platform (Kaggle.com), and OpenAI’s GPT-4 API was utilized for automated question answering. The program automatically recorded the number of words generated as well as the time consumed. The first agent responsible was categorization, which only ran once and did not participate in the answer-generation process. Therefore, time and words were not recorded for this agent. For the second and third agents, as their results mainly involved returning potential content from the guidelines and "True/False" answers, the words was not recorded as well.

### The direct-GPT(GPT-4)

The direct question-and-answer design was based on GPT-4, utilizing the same environment as GPT-agents. Within the design, all questions were posed within a "for" loop (similarly to in GPT-agents group), and GPT-4 directly generated responses (Fig. 1B). The process recorded all the content, including the time consumed and the word count of the generated answers.

### The medical guidelines

The references for TBI rehabilitation guidelines were obtained by searching a specialized Chinese database that collects all clinical guidelines and expert consensuses (Clinical Guidelines Network, https://guide.medlive.cn). Brain rehabilitation guidelines and standards were retrieved and thoroughly reviewed by a clinician (L.Z.Z.) with 14 years of clinical work experience. After clinical evaluation, the expert consensus^20^ that best aligns with Chinese TBI rehabilitation, was incorporated into the system to make it more comprehensive and inclusive of the content from traditional Chinese medicine.

### Question data collection

First, 300 real-world brain rehabilitation-related questions from doctor-patient interactions were collected from online sources. Two medical experts (L.Z.Z and Z.W), both with over 10 years of clinical experience, who worked at the same Grade A tertiary hospital, manually collected 300 Chinese brain injury rehabilitation-related questions from two open-source Chinese medical dialogue datasets (https://github.com/Toyhom/Chinese-medical-dialogue-data, datasets/ FreedomIntelligence/huatuo_knowledge_graph_qa) and one website (https://youlai.cn/). Each question is accompanied by an answer, and the responses to these questions are publicly available. These questions cover the various stages of brain injury rehabilitation. Second, we randomly selected 30 questions to ask and evaluate using a computer method (code:random.choice(list,30)).

The inclusion criteria were as follows: (1) questions related to brain rehabilitation; (2) answers by medical experts available; (3) publicly available question-and-answer pairs without involving personal privacy; and (4) no copyright restrictions. The exclusion criteria were as follows: (1) inadequate responses prompting further hospital visits; (2) questions focusing on severe complications in vital organs such as the heart or kidneys; (3) unanswered questions by doctors; and 4) questions violating medical ethics or Chinese laws in questions or answers.

### Evaluation for GPT-agents and GPT-4

The valuation team members included a chief physician (Z. J. F.), a senior physician (L. Z. Z.), and a nurse (X. R. Y.), all of whom had more than 10 years’ experience in clinical practice. The primary endpoint was better answers, whereas the secondary endpoint includes accuracy, completeness, explainability, and empathy.

First, a better evaluation of both answers (GPT-4 and GPT-agents) was required. Next, we evaluated the four sub-dimensions of accuracy, completeness, explainability, and empathy separately.

We developed a Likert scoring scale to evaluate the responses. To ensure accuracy, we referenced previous studies^36^ and adopted a continuous 5–0 rating system. The others were evaluated using a continuous 3–0 scale. A higher score signified strong agreement, whereas a score of 0 indicated strong disagreement (Supplementary Table 1).

### Statistical analysis

Categorical data of the primary endpoint are presented as the number of cases and their respective rates. Comparisons between groups were performed using the chi-square or Fisher’s exact tests. Other measurement data for the normal distribution are presented as means ± standard deviations, and comparisons between groups was conducted using two independent sample t-tests. The measurement data for skewed distribution are presented as medians and quartile ranges. The level of statistical significance was set at *p* < 0.05. All statistical analyses were performed using GraphPad software (version 8). The time consumed and word count were displayed using Matplotlib in Python 3.10.

### Supplementary Information

Supplementary Table 1.

Supplementary Table 2.

## Data Availability

The original data presented in the study are included in the article/supplementary material.
