# Supplementary material for: GPT-agents based on medical guidelines can improve the responsiveness and explainability of outcomes for traumatic brain injury rehabilitation
Source: Sci Rep. 2024 Apr 1;14:7626. doi: 10.1038/s41598-024-58514-9 (PMC10985066; doi:10.1038/s41598-024-58514-9)
Supplement: Supplementary file 1 — Supplementary Table 1. [file 41598_2024_58514_MOESM1_ESM.docx]

| **index** | **topic** | **Assessment content** | **Scoring reference** | **score** |
| --- | --- | --- | --- | --- |
| **1** | **Accuracy** | **The answer is very accurate.** | 5.0 for a correct answer,  4.0 for mostly consistent and non-conflicting answers,  3.0 for a few consistent and non-conflicting answers,  2.0 for mostly consistent but partially conflicting answers,  1.0 for mostly conflicting but a few consistent answers,  and 0 for completely conflicting answers. |  |
| **2** | **Completeness** | **The response is comprehensive and includes key clinical points.** | strong agreement：3；  strong disagreement:0 |  |
| **3** | **Explainability** | **The answer has excellent interpretability.** | strong agreement：3；  strong disagreement:0 |  |
| **4** | **Empathy** | **The response is empathetic, much like a family member.** | strong agreement：3；  strong disagreement:0 |  |

**The Likert scale for answer**
